# Hippocampal subfield radiomics reveals the link between inflammatory status and mild cognitive impairment in patients with non-dialysis chronic kidney disease

**DOI:** 10.3389/fnins.2026.1916421

**Published:** 2026-08-03

**Authors:** Zhichao Wang, Zhitao Zhu, Subo Zhang, Yu Zhang, Yun Liu, Wei Liu

**Affiliations:** 1 Department of Radiology, The Second People’s Hospital of Lianyungang, Lianyungang, Jiangsu, China; 2 Department of Radiology, The Affiliated People’s Hospital of Jiangsu University, Zhenjiang, Jiangsu, China; 3 Department of Radiology, Affiliated Hospital of Jiangsu University, Zhenjiang, Jiangsu, China

**Keywords:** chronic kidney disease, hippocampal subfields, inflammation, mild cognitive impairment, radiomics

## Abstract

**Objective:**

To investigate the associations among inflammatory status, hippocampal subfield radiomics phenotypes, and mild cognitive impairment (MCI) in patients with non-dialysis chronic kidney disease (CKD), and to construct an interpretable individualized risk prediction model.

**Methods:**

Patients with non-dialysis CKD stages 3–5 were included from a primary cohort of 212 patients, including 86 with CKD-MCI and 126 cognitively normal individuals, and an external validation cohort of 98 patients. Clinical data, inflammatory markers, and Montreal Cognitive Assessment (MoCA) data were collected. Based on 24 bilateral hippocampal subfields derived from high-resolution structural MRI, radiomics features were extracted and dimensionally reduced using mRMR and LASSO regression to construct a hippocampal radiomics score (Hip-Radscore). Mediation analysis was used to explore the association pathway among inflammation, Hip-Radscore, and MCI. A combined prediction model was constructed using the XGBoost algorithm and externally validated, with SHAP applied to interpret the model decision-making mechanism.

**Results:**

Patients with CKD-MCI exhibited an inflammatory status characterized by elevated hsCRP and SII levels. The Hip-Radscore constructed from eight core features, mainly texture features enriched in subfields such as CA1 and the subiculum, showed good performance in identifying MCI (AUC = 0.81), significantly outperforming the traditional hippocampal volume model (AUC = 0.67, *p* = 0.002). Mediation analysis showed that Hip-Radscore partially mediated the association between elevated hsCRP and lower MoCA scores; the indirect pathway through Hip-Radscore accounted for 39.4% of the total hsCRP–MoCA association. External validation confirmed that the XGBoost combined model integrating clinical inflammatory variables and Hip-Radscore achieved the best discriminative performance (AUC = 0.86), with good calibration and net benefit. SHAP analysis clearly showed that higher Hip-Radscore and hsCRP levels were the core features driving the model to predict a high risk of MCI.

**Conclusion:**

Elevated hsCRP was associated with hippocampal subfield radiomics abnormalities and cognitive decline in patients with non-dialysis CKD. Hip-Radscore may sensitively capture hippocampal tissue heterogeneity and may serve as a potential imaging intermediate phenotype linking inflammatory status to cognitive dysfunction. The interpretable XGBoost model integrating systemic inflammatory indicators and local radiomics features may provide a useful tool for identifying patients at high risk of CKD-related MCI.

## 1 Introduction

Chronic kidney disease (CKD) is characterized not only by progressive loss of renal function but also by a close association with cognitive decline. These two conditions may interact with each other, substantially reducing patients’ quality of life and affecting long-term prognosis (Aguilera et al., 2026; Lin and Hung, 2026). In patients with non-dialysis CKD stages 3–5, mild cognitive impairment (MCI) is relatively common and may involve multiple core cognitive domains at an early stage, including memory, executive function, attention, and information processing speed (Seidel et al., 2014). Because these patients have not yet initiated dialysis, subtle cognitive changes are easily overlooked in routine clinical management. However, early cognitive decline may affect disease self-management, treatment adherence, and long-term outcomes. Therefore, identifying sensitive, objective, and clinically interpretable biomarkers for the early detection of MCI in patients with non-dialysis CKD is of important clinical significance.

The pathogenesis of CKD-related MCI is complex and may involve multiple factors, including uremic toxin accumulation, cerebral small vessel disease, metabolic disturbance, anemia, oxidative stress, and chronic systemic inflammation (Xie et al., 2022; Frąk et al., 2024). Among these factors, chronic systemic inflammation is considered an important pathological link between renal function decline and brain structural abnormalities (Guo et al., 2023). Patients with CKD are chronically exposed to low-grade inflammatory activation. Inflammatory mediators may participate in central nervous system injury by disrupting the blood–brain barrier, damaging the vascular endothelium, impairing cerebral microvascular perfusion, and activating neuroglial cells (Wan et al., 2023). Clinically, peripheral indicators such as high-sensitivity C-reactive protein (hsCRP), systemic immune-inflammation index (SII), and neutrophil-to-lymphocyte ratio (NLR) can reflect systemic inflammatory burden from different dimensions. However, these indicators mainly characterize peripheral inflammatory status and cannot directly reveal the potential brain structural phenotype linking inflammatory burden to CKD-related MCI. In other words, it remains unclear whether a specific neuroimaging intermediate phenotype exists between systemic inflammation and CKD-related MCI.

The hippocampus is a key brain region involved in learning, memory, and cognitive integration, and it is also one of the structures that is vulnerable to early involvement in MCI (Davidson and Stevenson, 2024). Notably, the hippocampus is not a homogeneous structure. Its subfields have relatively specific anatomical connections and functional specialization. Subfields such as CA1, the subiculum, the dentate gyrus, and the presubiculum are particularly vulnerable to ischemia, inflammation, metabolic stress, and neurodegenerative changes (Bouwman et al., 2025). However, conventional MRI volumetric measurements mainly reflect macroscopic structural atrophy and are not sensitive to early or subtle microstructural heterogeneity within hippocampal subfields (Wang et al., 2026). Radiomics can extract high-dimensional intensity, texture, and spatial distribution features from routine structural MRI, enabling quantitative characterization of tissue heterogeneity and microstructural complexity at a level that is invisible to the naked eye (Shen et al., 2026). Therefore, hippocampal subfield-based radiomics phenotypes may be more sensitive than traditional volumetric indicators in reflecting early brain structural abnormalities associated with CKD-related MCI and may serve as an important imaging phenotype linking peripheral inflammatory status to cognitive decline.

However, existing neuroimaging studies on CKD-related cognitive dysfunction have mainly focused on global brain atrophy, white matter lesions, or whole hippocampal volume changes, with insufficient attention paid to microstructural heterogeneity at the hippocampal subfield level. More importantly, few studies have systematically evaluated the associations among systemic inflammation, hippocampal subfield radiomics phenotypes, and CKD-related MCI (Maki et al., 2023). This knowledge gap limits our understanding of the potential neuropathological processes underlying CKD-related MCI and hinders the development of early, precise, and interpretable risk identification strategies.

Based on this background, the present study focused on patients with non-dialysis CKD stages 3–5 and aimed to investigate the associations among systemic inflammatory status, hippocampal subfield radiomics phenotypes, and CKD-related MCI. Furthermore, we evaluated whether the hippocampal radiomics phenotype statistically mediated the association between inflammatory status and cognitive decline, and assessed its incremental value in the early identification of CKD-MCI. We hypothesized that hippocampal subfield radiomics phenotypes may serve as potential imaging intermediate phenotypes reflecting inflammation-related brain structural abnormalities and may help improve the accuracy and interpretability of MCI risk identification in patients with non-dialysis CKD.

## 2 Materials and methods

### 2.1 Study participants and clinical assessment

This study included patients with non-dialysis chronic kidney disease (CKD) stages 3–5. The primary cohort included 212 patients. This cohort was used for clinical characteristic comparison, hippocampal subfield radiomics feature selection, Hip-Radscore construction, and model training. An external validation cohort of 98 patients was used to evaluate the generalizability of the prediction model. All participants completed clinical data collection, laboratory examinations, neuropsychological assessment, and high-resolution structural MRI scanning. This retrospective study was approved by the Ethics Committee of The Affiliated People’s Hospital of Jiangsu University (K-20250715-Y). The requirement for written informed consent was waived because of the retrospective study design. The inclusion criteria were as follows: diagnosis of CKD stages 3–5 without dialysis treatment; age ≥18 years; ability to complete the Montreal Cognitive Assessment (MoCA) and MRI examination; and availability of complete clinical, laboratory, and imaging data. The exclusion criteria were as follows: a definite history of dementia, stroke, Parkinson’s disease, epilepsy, traumatic brain injury, or other central nervous system diseases that could affect cognitive function; severe psychiatric disorders; acute infection; active autoimmune disease; malignant tumor; contraindications to MRI; poor MRI image quality; or failure of hippocampal subfield segmentation.

Demographic data, CKD-related clinical indicators, comorbidities, and lifestyle information were collected from all participants. Demographic data included age, sex, body mass index (BMI), and years of education; CKD-related indicators included CKD stage, estimated glomerular filtration rate (eGFR), serum creatinine, blood urea nitrogen, albumin, and hemoglobin levels; comorbidities and lifestyle information included diabetes, hypertension, smoking history, and drinking history. CKD staging was classified according to the KDIGO guidelines. eGFR was calculated using the CKD-EPI equation. Inflammation-related indicators included high-sensitivity C-reactive protein (hsCRP), neutrophil-to-lymphocyte ratio (NLR), and systemic immune-inflammation index (SII). NLR was defined as the ratio of neutrophil count to lymphocyte count. SII was calculated as platelet count × neutrophil count/lymphocyte count. All participants underwent standardized MoCA assessment. For participants with fewer than 12 years of education, 1 point was added to the original score for correction. Cognitive grouping was performed based on the corrected MoCA total score. Participants with a corrected MoCA score ≥26 were defined as the cognitively normal group (CKD-NC). Participants with a corrected MoCA score <26 were defined as the mild cognitive impairment group (CKD-MCI) if their activities of daily living were basically preserved and they did not meet the diagnostic criteria for dementia according to DSM-5 or NIA-AA. The MoCA total score and scores for visuospatial and executive function, naming, attention, language, abstract thinking, delayed recall, and orientation were also recorded.

### 2.2 Imaging data acquisition and processing

All participants underwent high-resolution structural MRI scanning for hippocampal subfield segmentation and radiomics feature extraction. MRI examinations were performed using a 3.0 T magnetic resonance scanner. Three-dimensional T1-weighted structural images were acquired. During scanning, participants were required to keep their heads still to reduce motion artifacts. The main scanning parameters were as follows: repetition time (TR), 2,300 ms; echo time (TE), 2.3 ms; flip angle, 8°; and voxel size, 1.0 × 1.0 × 1.0 mm^3^. All raw images underwent quality control before subsequent analysis. Participants with obvious motion artifacts, structural distortion, or image quality insufficient for hippocampal subfield segmentation were excluded.

**Figure 1:**
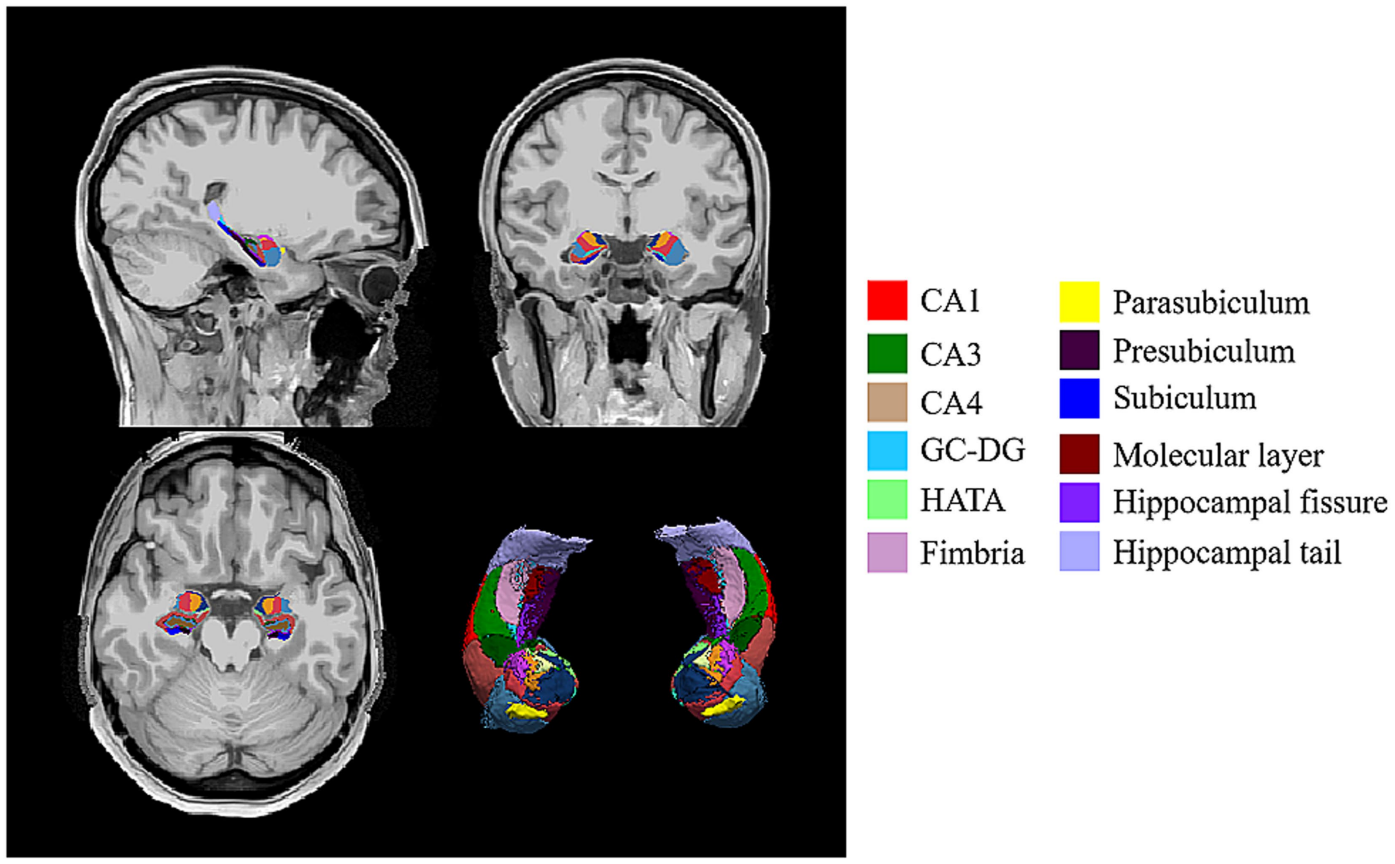
Schematic diagram of automatic hippocampal subfield segmentation. Based on high-resolution T1 structural images, FreeSurfer was used to automatically segment bilateral hippocampal subfields, and a total of 24 left and right hippocampal subfield ROIs were extracted for subsequent radiomics feature extraction. The figure shows the spatial distribution of hippocampal subfields in sagittal, coronal, axial, and three-dimensional reconstruction views.

Hippocampal subfield segmentation was performed using high-resolution T1 structural images. FreeSurfer software was used for standardized preprocessing of structural images. The preprocessing steps included skull stripping, intensity non-uniformity correction, spatial registration, tissue segmentation, and cortical and subcortical reconstruction. The FreeSurfer hippocampal subfield segmentation module was then used to automatically obtain bilateral hippocampal subfield regions of interest (ROIs). In this study, 24 bilateral hippocampal subfield ROIs were extracted for subsequent radiomics analysis (Figure 1). All segmentation results were manually inspected to confirm the accuracy of hippocampal subfield boundaries and spatial localization. Cases with segmentation failure or obvious anatomical errors were excluded.

Radiomics feature extraction was performed using PyRadiomics. Quantitative radiomics features were extracted from the T1 structural images within the 24 hippocampal subfield ROIs of each participant. To ensure consistency in feature extraction, all images were processed using unified preprocessing and gray-level discretization parameters before feature extraction. Ninety-three features were extracted from each hippocampal subfield ROI. These features included 18 first-order statistical features, 24 gray-level co-occurrence matrix (GLCM) features, 16 gray-level run length matrix (GLRLM) features, 16 gray-level size zone matrix (GLSZM) features, 14 gray-level dependence matrix (GLDM) features, and 5 neighboring gray tone difference matrix (NGTDM) features. Therefore, a total of 24 × 93 = 2,232 hippocampal subfield radiomics features were obtained for each participant. This was done to reduce the direct influence of hippocampal subfield volume differences on the radiomics model. The analysis focused on quantitative features reflecting local gray-level distribution, texture heterogeneity, and microstructural complexity. The primary cohort and the external validation cohort used the same hippocampal subfield segmentation, image preprocessing, and radiomics feature extraction procedures. This ensured feature comparability between cohorts. To evaluate the reproducibility of radiomics features, some participants were randomly selected for repeated feature extraction. The intraclass correlation coefficient (ICC) was used to assess feature stability.

### 2.3 Prediction model construction and interpretation

To construct a hippocampal subfield radiomics phenotype reflecting CKD-related mild cognitive impairment, radiomics feature selection was first performed in the primary cohort. All radiomics features were standardized before feature selection. This was done to reduce the influence of scale differences on model estimation. The Mann–Whitney U test was first used to screen features that differed between the CKD-MCI and CKD-NC groups. The minimum redundancy maximum relevance (mRMR) algorithm was then used to remove redundant features and retain features highly related to MCI status. Finally, least absolute shrinkage and selection operator (LASSO) regression was used for final feature selection. The optimal penalty parameter *λ* was determined by 10-fold cross-validation. Eight optimal hippocampal subfield radiomics features were selected to construct the hippocampal radiomics score (Hip-Radscore). Hip-Radscore was calculated using the selected features and their corresponding LASSO coefficients. The formula was as follows: Hip-Radscore = Σβ_i × X_i, where β_i represents the regression coefficient of the i-th selected feature, and X_i represents the standardized radiomics feature value.

To evaluate the incremental value of radiomics features compared with traditional structural indicators, a hippocampal subfield volume model was further constructed as a control model. Hippocampal subfield volume parameters output by FreeSurfer were corrected for intracranial volume and then included in model construction. To ensure a fair comparison, the volume model was constructed using the same primary cohort and evaluated using the same external validation cohort as the Hip-Radscore model. The volume model adopted a consistent modeling and validation strategy as the Hip-Radscore model to ensure comparability of performance between the two types of models based on identical study populations and validation procedures (Supplementary Figure S1). The extreme gradient boosting (XGBoost) algorithm was used to construct CKD-MCI prediction models. Three models were established according to predictor source. The clinical inflammation model included age, sex, years of education, eGFR, and hsCRP. The radiomics model included Hip-Radscore. The combined model included both clinical inflammatory predictors and Hip-Radscore. The models were trained in the primary cohort. Model parameters were optimized by cross-validation and parameter tuning to reduce overfitting and improve model stability. The final models were independently validated in the external validation cohort to evaluate their generalizability.

To improve the interpretability of the XGBoost combined model, SHAP (SHapley Additive exPlanations) was used to quantify the contribution of each predictor to model prediction. The SHAP summary plot displayed the overall importance ranking of predictors and their influence on the prediction direction of MCI. The SHAP dependence plot showed the relationship between key predictor values and predicted risk. The SHAP waterfall plot displayed the single-case prediction process and showed how different predictors jointly drove the model output. Based on the final XGBoost combined model, a locally deployed visual prediction interface was constructed. This interface allowed the input of age, sex, years of education, eGFR, hsCRP, and Hip-Radscore. It automatically generated individualized CKD-MCI prediction probabilities and risk stratification results. This interface was used only to demonstrate the potential clinical translation of the model. It was not used for model training, parameter tuning, or performance evaluation.

### 2.4 Statistical analysis

Continuous variables were first tested for normality. Normally distributed variables were expressed as mean ± standard deviation. Between-group comparisons were performed using the independent-samples t test. Non-normally distributed variables were expressed as median and interquartile range. Between-group comparisons were performed using the Mann–Whitney U test. Categorical variables were expressed as numbers and percentages. Between-group comparisons were performed using the χ^2^ test or Fisher’s exact test. Spearman correlation analysis was used to evaluate the relationships among inflammatory status, hippocampal subfield radiomics phenotypes, and cognitive function. Multiple correlation tests may increase the risk of type I error. Therefore, Bonferroni correction was applied. The corrected significance threshold was set at *p* < 0.002. Only inflammatory indicators that remained statistically significant after correction were included in subsequent mediation analysis.

A mediation model was constructed to investigate whether hippocampal subfield radiomics phenotypes mediated the relationship between inflammatory status and cognitive function. hsCRP was used as the independent variable. Hip-Radscore was used as the mediating variable, and MoCA total score was used as the dependent variable. Age, sex, years of education, diabetes, hypertension, and eGFR were included as covariates. The mediation effect was tested using 5,000 bootstrap resamples. The indirect effect and its 95% confidence interval were calculated. A mediation effect was considered significant when the 95% confidence interval of the indirect effect did not cross 0.

The discriminative performance of the prediction models was evaluated using receiver operating characteristic curves and the area under the curve. The 95% confidence intervals were also calculated. AUCs between different models were compared using the DeLong test. Bonferroni correction was applied according to the number of comparisons. Model calibration was evaluated using calibration curves and the Brier score. Clinical utility was assessed using decision curve analysis. All statistical tests were two-sided. *p* < 0.05 was considered statistically significant, except in analyses with multiple-comparison correction. For Bonferroni-corrected analyses, the corrected threshold was used.

## 3 Results

### 3.1 Clinical baseline characteristics

A total of 212 patients with non-dialysis chronic kidney disease (CKD stages 3–5) were included in the primary cohort. According to the Montreal Cognitive Assessment (MoCA) scores, patients were divided into the mild cognitive impairment group (MCI, *n* = 86) and the cognitively normal group (NC, *n* = 126). Compared with the NC group, patients in the MCI group were older and had fewer years of education (both *p* < 0.05). No significant differences were observed in other baseline characteristics between the two groups (all *p* > 0.05). Regarding inflammation-related indicators, hsCRP and SII levels were significantly higher in the MCI group than in the NC group (both *p* < 0.05). NLR showed an increasing trend in the MCI group, but the between-group difference was not statistically significant (*p* > 0.05; Table 1). The external validation cohort included 98 patients with non-dialysis CKD. Among them, 40 were classified as MCI and 58 as NC. Their demographic and clinical baseline characteristics were generally consistent with those of the primary cohort, as shown in Supplementary Table S1.

**Table 1:** Comparison of general characteristics in the primary cohort.

Variable	CKD-NC (*n* = 126)	CKD-MCI (*n* = 86)	t/Z/χ^2^	*p* value
Age, years	59.8 ± 8.7	65.1 ± 8.9	4.31	<0.001
Male sex, *n* (%)	72 (57.1)	46 (53.5)	0.27	0.602
BMI, kg/m^2^	24.2 ± 3.1	24.5 ± 3.3	0.68	0.501
Education, years	9.6 ± 3.4	7.8 ± 3.1	3.91	<0.001
CKD stage, *n* (%)			0.77	0.681
CKD stage 3	51 (40.5)	31 (36.0)		
CKD stage 4	46 (36.5)	34 (39.5)		
CKD stage 5	29 (23.0)	21 (24.4)		
eGFR, mL/min/1.73 m^2^	31.8 ± 12.6	30.1 ± 12.1	0.98	0.329
Serum creatinine, μmol/L	226.5 (164.3–319.8)	239.7 (171.6–335.2)	0.81	0.417
Blood urea nitrogen, mmol/L	12.6 ± 4.8	13.2 ± 5.1	0.88	0.382
Albumin, g/L	38.4 ± 4.2	37.7 ± 4.5	1.17	0.245
Hemoglobin, g/L	112.8 ± 18.5	109.6 ± 17.9	1.25	0.214
Diabetes mellitus, n (%)	48 (38.1)	37 (43.0)	0.52	0.471
Hypertension, *n* (%)	91 (72.2)	67 (77.9)	0.87	0.351
Smoking history, *n* (%)	36 (28.6)	29 (33.7)	0.63	0.426
Alcohol history, *n* (%)	31 (24.6)	25 (29.1)	0.53	0.468
hsCRP, mg/L	2.4 (1.3–4.5)	4.1 (2.2–7.0)	4.21	<0.001
NLR	2.32 (1.72–3.08)	2.61 (1.91–3.38)	1.72	0.086
SII, ×10^9^/L	518.6 (389.4–702.1)	632.7 (461.8–846.5)	2.86	0.004
MoCA score	26.8 ± 1.2	21.9 ± 2.5	16.99	<0.001

Data are presented as mean ± standard deviation, median (interquartile range), or number (percentage), as appropriate. BMI, body mass index; CKD, chronic kidney disease; eGFR, estimated glomerular filtration rate; hsCRP, high-sensitivity C-reactive protein; NLR, neutrophil-to-lymphocyte ratio; SII, systemic immune-inflammation index; MoCA, Montreal Cognitive Assessment.

### 3.2 Hippocampal subfield radiomics feature selection and Hip-Radscore construction

**Figure 2:**
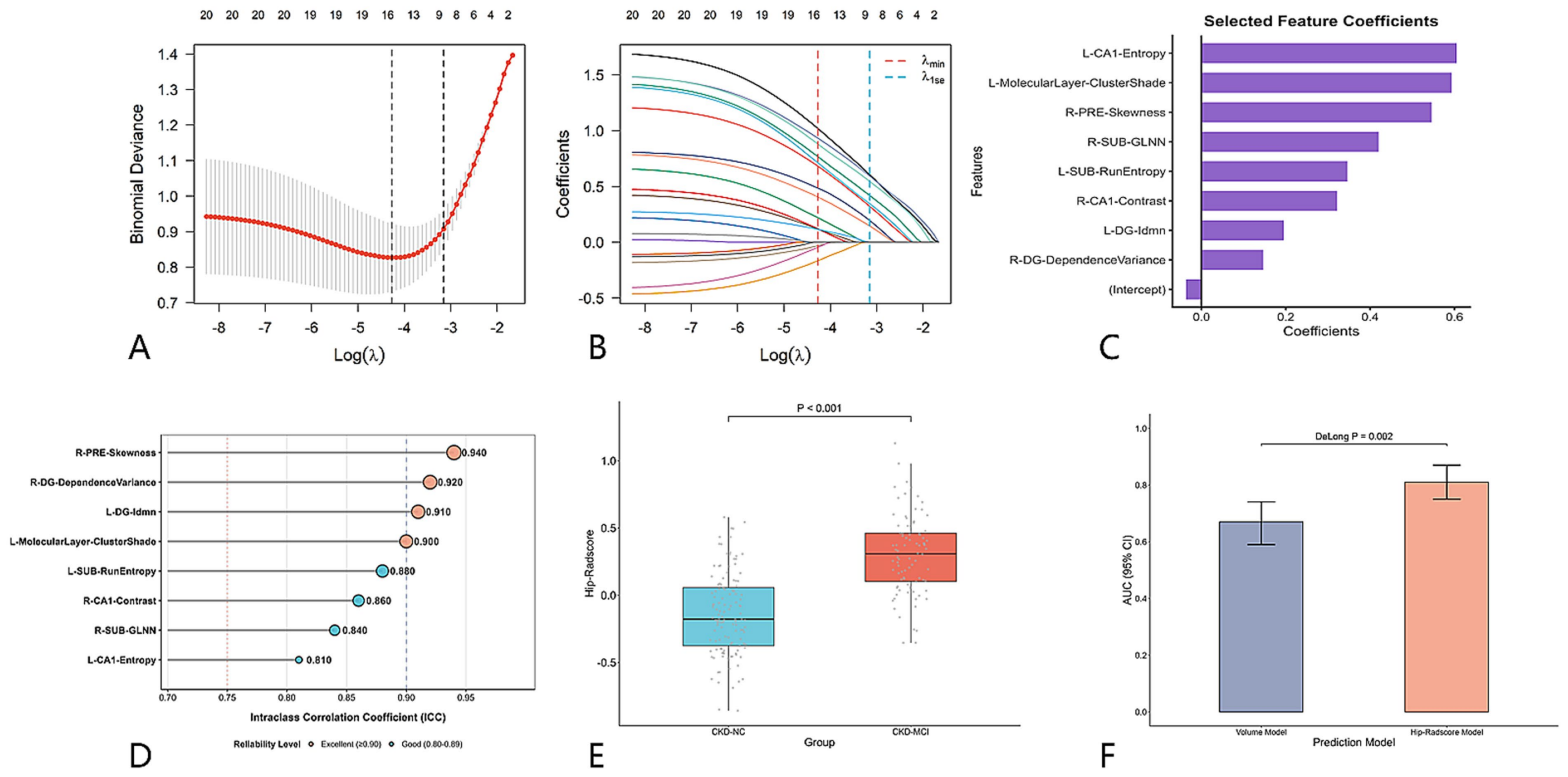
Hippocampal subfield radiomics feature selection and Hip-Radscore construction. **(A)** Ten-fold cross-validated LASSO regression was used to determine the optimal penalty parameter *λ*; **(B)** The LASSO coefficient path plot shows the shrinkage process of candidate features with changes in *λ*; **(C)** The eight finally selected hippocampal subfield radiomics features and their regression coefficients; **(D)** The ICC distribution of the selected features, showing good to excellent reproducibility; **(E)** Hip-Radscore was significantly higher in the CKD-mild group than in the CKD-NC group; **(F)** The discriminative performance of the Hip-Radscore model was superior to that of the hippocampal subfield volume model.

A total of 2,232 hippocampal subfield radiomics features were extracted from high-resolution structural MRI. Feature selection was performed using the Mann–Whitney U test, mRMR, and 10-fold cross-validated LASSO regression. Eight optimal features were finally identified to construct the Hip-Radscore. These features included L-CA1-Entropy, R-CA1-Contrast, L-SUB-RunEntropy, R-SUB-GLNN, L-DG-Idmn, R-DG-DependenceVariance, L-MolecularLayer-ClusterShade, and R-PRE-Skewness (Figures 2A–C). The selected features were mainly located in CA1, the subiculum, the dentate gyrus, the hippocampal molecular layer, and the presubiculum. Texture features accounted for 7 of the 8 selected features. The ICC of the selected features ranged from 0.80 to 0.93. The Hip-Radscore was significantly higher in the MCI group than in the NC group (0.26 ± 0.33 vs. −0.12 ± 0.29, *p* < 0.001). The Hip-Radscore model showed good discriminative performance (AUC = 0.81, 95% CI: 0.75–0.87). Its performance was significantly better than that of the hippocampal subfield volume model (AUC = 0.67, 95% CI: 0.59–0.74; DeLong *p* = 0.002; Figures 2D–F).

### 3.3 Associations among inflammatory status, radiomics phenotype, and cognitive function

**Figure 3:**
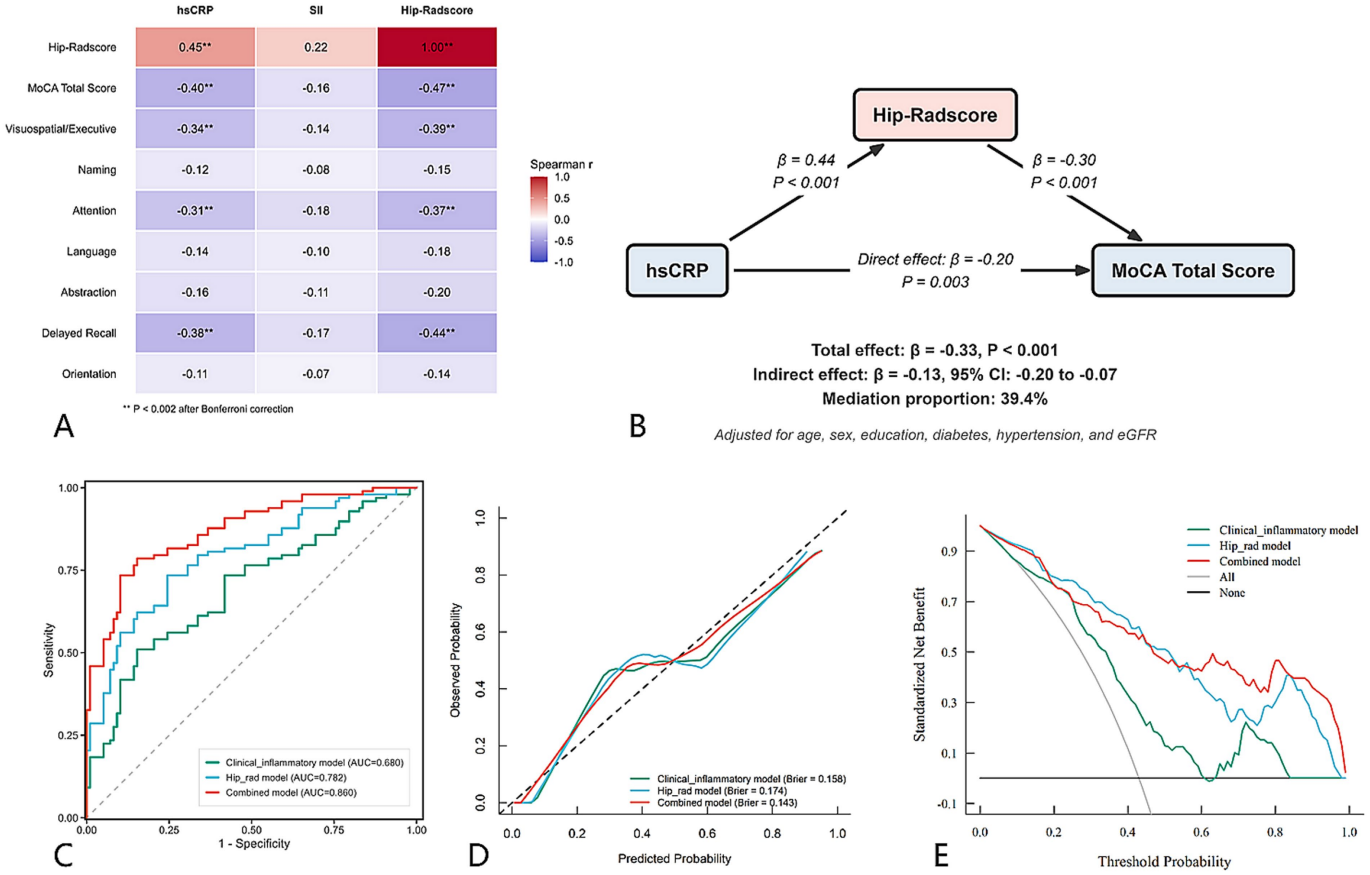
Analysis of inflammatory status, hippocampal radiomics phenotype, and CKD-MCI prediction models. **(A)** Correlation heatmap of hsCRP, SII, Hip-Radscore, MoCA total score, and scores of each cognitive domain; **(B)** Mediation effect model of Hip-Radscore between hsCRP and MoCA total score; **(C)** ROC curves of the clinical inflammation model, radiomics model, and combined model in the external validation cohort; **(D)** Calibration curves of the three models; **(E)** Decision curve analysis showing that the combined model had higher net benefit across a relatively wide range of threshold probabilities. The symbol ** indicates *P* < 0.002 after Bonferroni correction, as shown in Figure 3A.

For multiple correlation analyses, the Bonferroni method was used for multiple-comparison correction. The corrected significance threshold was *p* = 0.002. After correction, hsCRP showed a significant positive correlation with Hip-Radscore (*r* = 0.45, *p* < 0.001). By contrast, the correlation between SII and Hip-Radscore did not reach the corrected significance threshold (*r* = 0.22, *p* = 0.028). hsCRP was significantly negatively correlated with MoCA total score, delayed recall, attention, and visuospatial and executive function scores (*r* = −0.31 to −0.40, all *p* < 0.001). Similarly, Hip-Radscore was significantly negatively correlated with these cognitive indicators (*r* = −0.37 to −0.47, all *p* < 0.001; Figure 3A).

### 3.4 Mediation effect of the hippocampal subfield radiomics phenotype

To explore the potential link between inflammatory status and cognitive function, a mediation model was constructed. In this model, hsCRP was used as the independent variable, Hip-Radscore as the mediator, and MoCA total score as the dependent variable. Age, sex, years of education, diabetes, hypertension, and eGFR were included as covariates. The results showed that hsCRP was significantly positively associated with Hip-Radscore (*β* = 0.44, *p* < 0.001). Hip-Radscore was significantly negatively associated with MoCA total score (*β* = −0.30, *p* < 0.001). The total effect of hsCRP on MoCA total score was significant (*β* = −0.33, *p* < 0.001). The direct effect remained significant after including Hip-Radscore in the model (*β* = −0.20, *p* = 0.003). Bootstrap testing confirmed a significant mediation effect of Hip-Radscore. The indirect effect was −0.13, with a 95% CI of −0.20 to −0.07. The indirect pathway through Hip-Radscore accounted for 39.4% of the total hsCRP–MoCA association (Figure 3B).

### 3.5 Model performance analysis

The predictive performance of three XGBoost models was compared. These models included the clinical inflammation model, the radiomics model, and the combined model. The clinical inflammation model included age, sex, years of education, eGFR, and hsCRP. The radiomics model included Hip-Radscore. The combined model included both clinical inflammatory predictors and Hip-Radscore. In the external validation cohort, the combined model showed the best discriminative ability. Its AUC was 0.86 (95% CI: 0.81–0.94). This was higher than the AUC of the clinical inflammation model (AUC = 0.68, 95% CI: 0.65–0.78) and the radiomics model (AUC = 0.78, 95% CI: 0.70–0.87; Figure 3C). The DeLong test showed that the combined model performed significantly better than the clinical inflammation model (*p* < 0.001) and the radiomics model (*p* = 0.004). These differences remained significant after Bonferroni correction. The calibration curve showed good predictive consistency for the combined model (Brier = 0.132; Figure 3D). Decision curve analysis showed that the combined model provided higher net benefit across a broad range of threshold probabilities (Figure 3E).

### 3.6 SHAP model interpretation analysis

**Figure 4:**
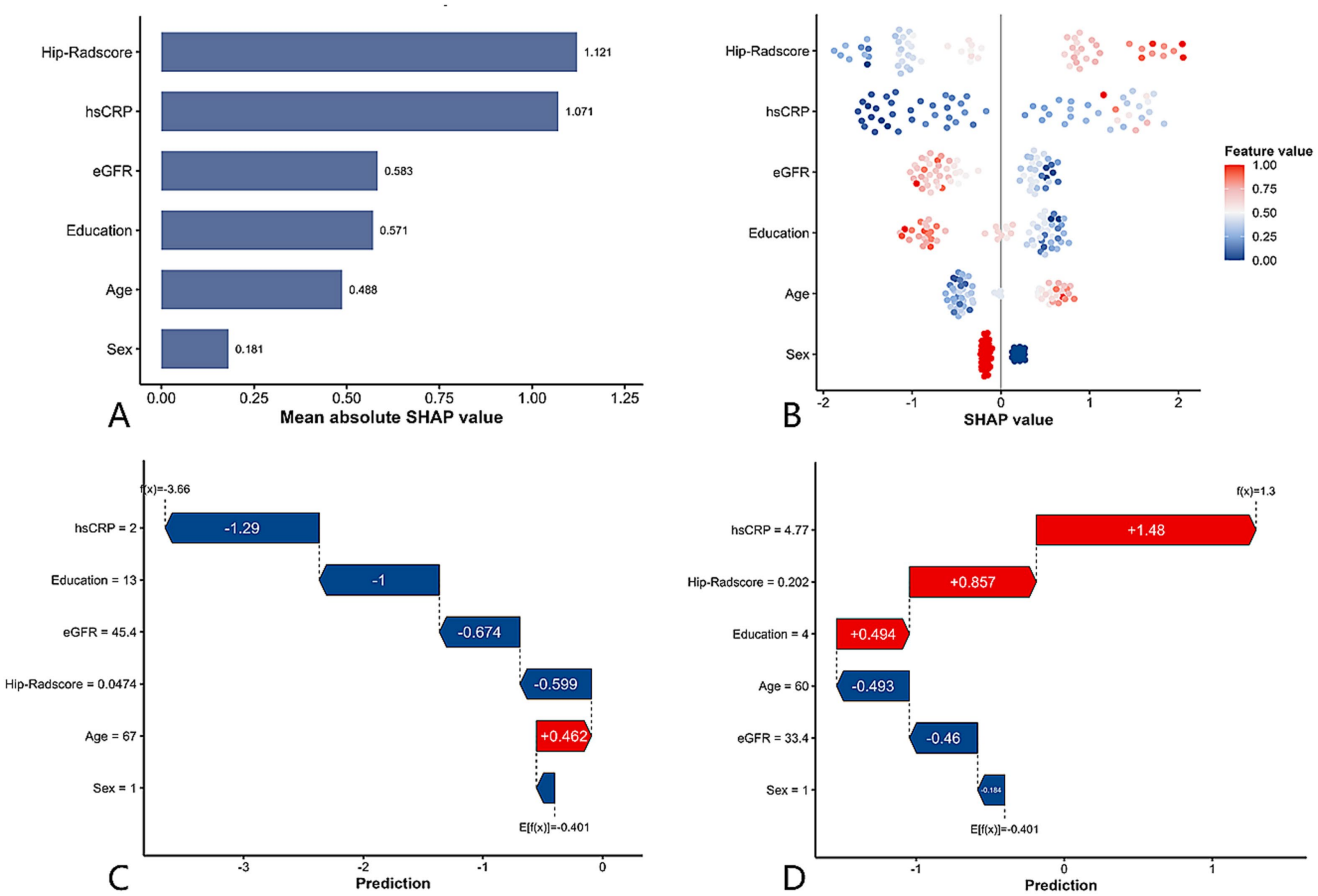
SHAP interpretation analysis of the XGBoost combined model. **(A)** Mean absolute SHAP values of each feature, showing the average contribution of each feature to model prediction. Hip-Radscore contributed the most, followed by hsCRP, eGFR, years of education, age, and sex. **(B)** SHAP summary plot showing the direction and magnitude of each feature’s contribution. The vertical gray line at SHAP value = 0 indicates the baseline model output. Points on the right side indicate features that increase the predicted probability of CKD-MCI, whereas points on the left side indicate features that decrease the predicted probability. The color gradient represents normalized feature values, with red indicating higher values and blue indicating lower values. **(C,D)** SHAP waterfall plots of two representative individuals. Red bars indicate features that increase the predicted risk of CKD-MCI, whereas blue bars indicate features that decrease the predicted risk. The length of each bar represents the magnitude of the feature contribution to the individual prediction.

SHAP analysis showed that Hip-Radscore had the largest contribution to the XGBoost combined model, followed by hsCRP, eGFR, years of education, age, and sex (Figure 4A). In the SHAP summary plot, the gray line at SHAP value = 0 represented no directional contribution to the prediction. Features distributed on the right side increased the predicted probability of CKD-MCI, whereas those on the left side decreased it. The color gradient indicated normalized feature values, with red representing higher values and blue representing lower values. Higher Hip-Radscore and hsCRP values were mainly located on the positive SHAP side, indicating increased CKD-MCI risk. By contrast, higher eGFR and more years of education were mainly located on the negative SHAP side, suggesting reduced predicted risk (Figure 4B and Supplementary Figure S2). Waterfall plots further illustrated individual-level prediction. Red bars indicated features that increased the predicted risk, and blue bars indicated features that decreased it; longer bars represented larger contributions. In the low-risk case, lower hsCRP, higher education level, higher eGFR, and lower Hip-Radscore shifted the prediction toward lower risk (Figure 4C). In the high-risk case, higher hsCRP, higher Hip-Radscore, and lower education level shifted the prediction toward higher risk, partly offset by eGFR and age (Figure 4D). The locally deployed visualization interface is shown in Supplementary Figure S3.

## 4 Discussion

These findings suggest that Hip-Radscore may serve as a potential imaging intermediate phenotype associated with inflammation-related cognitive decline in patients with non-dialysis CKD. The results showed that patients with CKD-MCI had an inflammatory status characterized by elevated hsCRP. hsCRP was also robustly associated with cognitive decline and Hip-Radscore. Compared with traditional volumetric indicators, Hip-Radscore captured hippocampal microstructural heterogeneity more sensitively. It also showed better performance in identifying CKD-MCI. In addition, Hip-Radscore partially mediated the association between hsCRP and MCI. These findings suggest that Hip-Radscore may serve as an imaging intermediate phenotype. In terms of clinical prediction, external validation confirmed that the XGBoost model integrating clinical inflammation and Hip-Radscore had the best predictive performance. SHAP analysis further showed that both hsCRP and Hip-Radscore were core contributors to model prediction. Overall, this study identified an association pattern consistent with a potential pathway of “systemic inflammation—hippocampal subfield radiomics abnormalities—cognitive decline.” However, given the cross-sectional design, this pathway should be interpreted as a hypothesis-generating framework rather than evidence of a causal cascade. It also provides a quantifiable and interpretable imaging marker for early cognitive risk assessment in patients with non-dialysis CKD.

Chronic low-grade inflammation is an important pathological basis for CKD progression and its neurological complications. With declining renal function, uremic toxin accumulation, oxidative stress, and endothelial dysfunction may activate systemic inflammation. These processes may disrupt blood–brain barrier integrity and induce central neuroinflammation (Wojtaszek et al., 2021). In this study, hsCRP and SII levels were significantly elevated in patients with CKD-MCI, suggesting that increased inflammatory burden may be involved in MCI in non-dialysis CKD. Among the inflammatory indicators, hsCRP showed the most robust association with MCI. Although SII was significantly elevated in the CKD-MCI group, its correlation with Hip-Radscore did not survive multiple-comparison correction. NLR only showed an increasing trend. In contrast, hsCRP was positively correlated with Hip-Radscore and negatively correlated with MoCA total score and multiple cognitive subdomains. These findings suggest that hsCRP may more sensitively reflect the inflammatory burden associated with brain structural abnormalities and cognitive decline in CKD. Age is another important factor in cognitive decline. Cognitive decline becomes more common in individuals aged 65 years and older, even without CKD. In this study, patients with CKD-MCI were older than those with normal cognition. Thus, age may partly contribute to the observed cognitive differences. To reduce this confounding effect, age was included as a covariate in the mediation analysis and as a predictor in the XGBoost models. SHAP analysis showed that age contributed to prediction, but its contribution was lower than those of Hip-Radscore and hsCRP. This suggests that the hsCRP–Hip-Radscore–cognition association was not fully explained by age alone. CKD-related MCI is difficult to explain solely by aging, renal function decline, or traditional vascular risk factors. Persistently elevated inflammatory burden may contribute to cognitive network disruption by aggravating microvascular injury, activating neuroglial cells, weakening synaptic plasticity, and increasing hippocampal metabolic vulnerability (Bolignano et al., 2025; Xu et al., 2025). Therefore, elevated hsCRP may reflect an upstream inflammatory environment associated with hippocampal microstructural alterations and cognitive decline. Nevertheless, residual age-related effects cannot be completely excluded, and future longitudinal studies with age-stratified analyses are needed.

The hippocampus is a key structure for memory processing and cognitive integration. It is also vulnerable to CKD-related metabolic abnormalities, microvascular injury, and inflammatory responses. Traditional hippocampal volume analysis mainly reflects macroscopic atrophy. It is less sensitive to early or subtle changes in tissue heterogeneity (Uchida et al., 2026; Aziz et al., 2026). In this study, radiomics features were extracted from 24 bilateral hippocampal subfields. These features were used to construct Hip-Radscore. The results showed that Hip-Radscore had good ability to identify CKD-MCI. Its performance was significantly better than that of the hippocampal subfield volume model. These findings suggest that hippocampal radiomics can provide microstructural information beyond traditional volumetric measurements (Zheng et al., 2025). The selected features were mainly distributed in CA1, the subiculum, the dentate gyrus, the molecular layer, and the presubiculum. Most selected features were texture features. These features reflect local gray-level distribution, texture complexity, and signal heterogeneity (Meng et al., 2024). They may correspond to subtle tissue changes related to inflammation, oxidative stress, microvascular perfusion abnormalities, and neuronal metabolic dysfunction in CKD. CA1 and the subiculum are important nodes of the hippocampal output pathway. They are particularly sensitive to ischemia, inflammation, and metabolic stress. The dentate gyrus is closely related to pattern separation and memory encoding. Abnormalities in this region may be associated with early memory decline (Li et al., 2025). Therefore, elevated Hip-Radscore may represent increased hippocampal subfield microstructural heterogeneity and enhanced network vulnerability in patients with CKD-MCI. Notably, Hip-Radscore was negatively correlated with MoCA total score and multiple cognitive domain scores in this study. This indicates that Hip-Radscore is not merely a classification feature. It is also continuously associated with the severity of cognitive decline. Compared with macroscopic volumetric indicators, hippocampal radiomics can more sensitively characterize early brain structural abnormalities in CKD-related MCI. It may also provide a quantifiable intracerebral phenotype that reflects the association between inflammatory status and cognitive function.

The mediation analysis showed that Hip-Radscore statistically mediated the association between elevated hsCRP and lower MoCA scores. This finding supports a potential association pathway of “systemic inflammation—hippocampal radiomics abnormalities—cognitive decline.” However, this result should be interpreted cautiously because mediation analysis based on cross-sectional data cannot establish temporal or causal relationships. CKD patients are chronically exposed to uremic toxin accumulation, oxidative stress, and endothelial dysfunction. These pathological conditions may activate peripheral inflammation. They may also affect cerebral microvascular homeostasis, blood–brain barrier integrity, and neuroglial activation. Through these processes, CKD may contribute to changes in gray-level distribution and texture complexity in hippocampal subfields (Yu et al., 2022; Wang et al., 2023). The microstructural heterogeneity captured by Hip-Radscore may reflect the imaging manifestation of inflammation-related brain injury on structural MRI. However, the partial mediating effect also indicates that other mechanisms may be involved. These mechanisms may include white matter injury, anemia, metabolic disturbance, and vascular risk factors (Miwa and Toyoda, 2022; Blasco-Colmenares et al., 2024; Pépin et al., 2023). The XGBoost combined model outperformed the clinical inflammation model and the radiomics model in external validation. This suggests that clinical inflammatory predictors and Hip-Radscore have complementary predictive value. They may reflect both the systemic pathological environment and the brain structural response. SHAP analysis showed that Hip-Radscore and hsCRP were the main predictive contributors. The locally deployed visualization interface can output individualized CKD-MCI prediction probability and risk stratification. This interface provides a preliminary prototype for clinical translation. However, prospective validation is still needed.

This study still has certain limitations. First, this study used a cross-sectional observational design; therefore, causal relationships among systemic inflammation, hippocampal subfield radiomics abnormalities, and MCI cannot be established. Although the mediation analysis suggested a potential statistical pathway linking elevated hsCRP, increased Hip-Radscore, and lower MoCA scores, this result should not be interpreted as evidence of causality. Longitudinal studies with repeated inflammatory measurements, serial MRI examinations, and prospective cognitive follow-up are needed to determine the temporal sequence and causal relationships among these factors. Second, although an external validation cohort was included, the sample size remains limited. The stability and generalizability of the model should be further confirmed in larger multicenter cohorts. Third, radiomics features may be affected by scanning parameters, segmentation accuracy, and preprocessing procedures. Therefore, standardized imaging acquisition and processing procedures should be emphasized in future studies.

## Data Availability

The raw data supporting the conclusions of this article will be made available by the authors, without undue reservation.

## Supplementary material

SUPPLEMENTARY FIGURE S1 Feature selection process of the hippocampal subfield volume model. **(A)** LASSO regression coefficient path plot for hippocampal subfield volume features. Each colored curve represents the coefficient trajectory of one hippocampal subfield volume feature as the penalty parameter λ changes. The dashed vertical lines indicate λ_min and λ_1se selected by ten-fold cross-validation. **(B)** Ten-fold cross-validation curve used to determine the optimal λ value. Red dots represent the mean binomial deviance at each λ value, and gray error bars represent the standard error. **(C)** The finally selected hippocampal subfield volume features and their regression coefficients used to construct the volume control model. L- and R- indicate left and right hippocampal subfields, respectively.

SUPPLEMENTARY FIGURE S2 SHAP dependence plots of each predictor in the XGBoost combined model. The SHAP dependence plots show the effects of changes in Hip-Radscore, hsCRP, eGFR, years of education, age, and sex on the predicted risk of CKD-MCI.

SUPPLEMENTARY FIGURE S3 Locally deployed CKD-MCI risk prediction and SHAP interpretation interface. This interactive interface was constructed based on the final XGBoost combined model. It supports the input of Hip-Radscore, hsCRP, eGFR, years of education, age, and sex, and outputs individualized CKD-MCI prediction probability and dynamic SHAP waterfall plots, which are used to display model prediction results and the contribution direction of each predictor.
