# Supplementary material for: Hippocampal subfield radiomics reveals the link between inflammatory status and mild cognitive impairment in patients with non-dialysis chronic kidney disease
Source: Front Neurosci. 2026 Aug 3;20:1916421. doi: 10.3389/fnins.2026.1916421 (PMC13478132; doi:10.3389/fnins.2026.1916421)
Supplement: Supplementary file 1 [file Supplementary_file_1.DOCX]

| **Supplementary Table 1. Comparison of General Characteristics in the Validation Cohort** | | | | |
| --- | --- | --- | --- | --- |
| **Variable** | **CKD-NC**  **(n = 58)** | **CKD-MCI**  **(n = 40)** | t/Z/χ² | **P value** |
| Age, years | 60.6 ± 8.4 | 64.8 ± 8.6 | 2.41 | 0.018 |
| Male sex, n (%) | 33 (56.9) | 21 (52.5) | 0.19 | 0.666 |
| BMI, kg/m² | 24.0 ± 3.0 | 24.4 ± 3.2 | 0.63 | 0.529 |
| Education, years | 9.4 ± 3.2 | 7.9 ± 3.0 | 2.31 | 0.023 |
| CKD stage, n (%) |  |  | 0.40 | 0.819 |
| CKD stage 3 | 24 (41.4) | 15 (37.5) |  |  |
| CKD stage 4 | 21 (36.2) | 16 (40.0) |  |  |
| CKD stage 5 | 13 (22.4) | 9 (22.5) |  |  |
| eGFR, mL/min/1.73 m² | 32.4 ± 12.2 | 30.7 ± 11.8 | 0.69 | 0.494 |
| Serum creatinine, μmol/L | 219.8  (158.5–306.4) | 234.1  (169.2–326.7) | 0.75 | 0.456 |
| Blood urea nitrogen, mmol/L | 12.3 ± 4.5 | 13.0 ± 4.9 | 0.73 | 0.471 |
| Albumin, g/L | 38.6 ± 4.0 | 37.9 ± 4.4 | 0.82 | 0.413 |
| Hemoglobin, g/L | 113.5 ± 17.6 | 110.1 ± 18.2 | 0.93 | 0.356 |
| Diabetes mellitus, n (%) | 22 (37.9) | 17 (42.5) | 0.21 | 0.649 |
| Hypertension, n (%) | 42 (72.4) | 31 (77.5) | 0.32 | 0.569 |
| Smoking history, n (%) | 17 (29.3) | 14 (35.0) | 0.35 | 0.552 |
| Alcohol history, n (%) | 15 (25.9) | 12 (30.0) | 0.20 | 0.655 |
| hsCRP, mg/L | 2.5 (1.4–4.6) | 3.8 (2.1–6.6) | 2.76 | 0.006 |
| NLR | 2.28 (1.69–3.02) | 2.56 (1.88–3.31) | 1.46 | 0.144 |
| SII, ×10⁹/L | 511.2  (381.6–689.5) | 621.4  (448.3–816.2) | 2.12 | 0.034 |
| MoCA score | 26.7 ± 1.1 | 22.1 ± 2.4 | 11.42 | <0.001 |
| **Note:** Data are presented as mean ± standard deviation, median (interquartile range), or number (percentage), as appropriate. BMI, body mass index; CKD, chronic kidney disease; eGFR, estimated glomerular filtration rate; hsCRP, high-sensitivity C-reactive protein; NLR, neutrophil-to-lymphocyte ratio; SII, systemic immune-inflammation index; MoCA, Montreal Cognitive Assessment. | | | | |
